# Evaluation of early myocardial damage in systemic sclerosis (SSc): a cardiovascular magnetic resonance study

**DOI:** 10.1186/1532-429X-17-S1-P264

**Published:** 2015-02-03

**Authors:** Nicola Galea, Marco Francone, Giovanni Barchetti, Amelia C Trombetta, Edoardo Rosato, Iacopo Carbone

**Affiliations:** Department of Radiological Sciences, Oncology and Pathology, University of Rome “Sapienza”, Rome, Italy; Department of Clinical Medicine, Clinical Immunology Unit-Scleroderma Center, University of Rome “Sapienza”, Rome, Italy

## Background

Cardiac involvement in systemic sclerosis (SSc) is mainly characterised by diffuse myocardial fibrosis and impairment of the microcirculation; even if clinically silent, it is increasingly recognized to be responsible for higher morbidity and mortality rate. Cardiovascular magnetic resonance (CMR) is the most accurate non-invasive method able to detect myocardial inflammation and fibrosis, even though traditional CMR techniques such as T2-weighted and late gadolinium enhancement (LGE) are inaccurate to assess diffuse myocardial disease.

Our aim was to investigate diffuse cardiac damage and perfusion abnormalities in asymptomatic SSc patients without known cardiac disease, using newer non-invasive imaging technique, such as T1 mapping and extracellular volume fraction (ECV), in pre-clinical stage.

## Methods

30 SSc patients (20 females, mean age: 35y) and 10 healthy controls (6 females, mean age: 34y) underwent CMR at 1.5 T unit (Magnetom Avanto, Siemens). CMR protocol included: cineMR sequences, STIR T2w, MOLLI T1 mapping pre-contrast. LGE imaging and MOLLI sequence for ECV quantification were acquired 10-15 min after administration of a bolus 0.1 mmol/Kg of Gadobenate dimeglumine (Gd-BOPTA)

## Results

LGE imaging was found to be positive in 7 SSc patients (23%) but none of controls. T2 weighted imaging found focal areas of inflammation in 3 SSc patients (10%), being negative in all controls. Native myocardial T1 values were significantly higher in SSc patients compared to controls (1043±37 vs. 980±33 p<0.01) and a significant difference in ECV expansion was found as well (30% vs. 23%, p<0.05). Notably, T1 values and ECV did not correlate with the presence of LGE but they were associated to higher disease activity and severity.

## Conclusions

SSc patients with no cardiac symptoms and preserved left ventricular size and function have nonetheless cardiac involvement, which can be detected with conventional T2 weighted and LGE imaging. Newer techniques are much more sensible in identifying diffuse cardiac pathology, since the elevated T1 values and expanded ECV fractions found in patients are probably due to the underlying low-grade inflammation and diffuse fibrosis.

